# Tubby is required for trafficking G protein-coupled receptors to neuronal cilia

**DOI:** 10.1186/2046-2530-1-21

**Published:** 2012-11-01

**Authors:** Xun Sun, James Haley, Oleg V Bulgakov, Xue Cai, James McGinnis, Tiansen Li

**Affiliations:** 1 Neurobiology Neurodegeneration and Repair Laboratory (N-NRL), National Eye Institute, MSC0610, 6 Center Drive, Bethesda, MD, 20892, USA; 2 Department of Ophthalmology, Dean A. McGee Eye Institute, Oklahoma University Health Sciences Center, Oklahoma City, 73104, USA

**Keywords:** Cilia, Neuronal cilia, Connecting cilia

## Abstract

**Background:**

Tubby is the founding member of the tubby-like family of proteins. The naturally occurring *tubby* mutation in mice causes retinitis pigmentosa, hearing loss and obesity. Tubby has been proposed to function as an accessory factor in ciliary trafficking. We directly examined a role for tubby in ciliary trafficking *in vivo*.

**Methods:**

We used immunofluoresence labeling to examine the subcellular localization of rhodopsin, somatostatin receptor 3 (SSTR3) and melanin concentrating hormone receptor 1 (MCHR1), all of which are G protein-coupled receptors (GPCR), in the retina and brain of wild type (WT) and *tubby* mutant mice.

**Results:**

In *tubby* mouse retina, rhodopsin is not fully transported across the connecting cilia to the outer segments with ensuing photoreceptor degeneration. In the *tubby* mouse brain, SSTR3 and MCHR1 fail to localize at the neuronal primary cilia in regions where these receptors play critical roles in neural signaling. The *tubby* mutant does not manifest a generalized defect in ciliogenesis or protein trafficking.

**Conclusions:**

Tubby plays a critical role in trafficking select GPCRs to the cilia. This role is reminiscent of tubby-like proteins 1 and 3, which have been proposed to facilitate trafficking of rhodopsin and select GPCRs in photoreceptors and the developing neural tube, respectively. Thus tubby-like proteins may be generally involved in transciliary trafficking of GPCRs.

## Background

The tubby-like proteins are defined by a highly conserved carboxyl terminal half of their primary sequence known as the tubby signature domain [1,2]. This family of proteins includes the prototype tubby, and TULP1, 2 and 3, for tubby-like proteins 1, 2 and 3 [3-5]. Other than members of the tubby family, search of sequence databases reveals no significant homology with known proteins or functional motifs. The tubby gene (*Tub*) was originally discovered by way of a spontaneously arisen obesity model in mice, and other members of the family were subsequently identified by homology cloning [3]. Mutations in human *TULP1* are a cause of retinitis pigmentosa [6]. Loss of TULP1 function in mice replicates this rapid photoreceptor degeneration phenotype [7,8]. Prior to photoreceptor degeneration in the mouse retina, pronounced ectopic distribution of rhodopsin is apparent indicating a defect in trafficking across the connecting cilia to reach their normal destination, the outer segments [9]. Loss of TULP3 function in mice leads to neural tube patterning defects and embryonic lethality [10], and the cellular basis can be traced to a failure of Hedgehog signaling due to defective ciliary trafficking [11]. Little is known about *Tulp2*, but its Chlamydomonas ortholog was identified as one of strongly induced genes during flagellar regeneration [12] and it was also reported as a candidate gene for human obesity in linkage analysis [13]. The tubby signature domain binds polyphosphorylated phosphatidylinositol [14], but their N-terminal domain is much more diverse. In the best characterized example, the TULP3 N-terminal domain binds to the IFT-A complex, which is part of the essential cellular machinery for ciliary transport, through a short conserved motif. In cultured cells, TULP3 facilitates membrane receptor trafficking to primary cilia. Thus it serves as bipartite bridges through their phosphoinositide-binding tubby domain and N-terminal IFT-binding motif, coordinating multiple signaling pathways including membrane receptor trafficking [15].

Originally designated *rd5*[16], the spontaneously arisen *tubby* mouse mutant manifests retinal degeneration, hearing loss and obesity, a tripartite phenotype that resembles mouse models of Bardet-Biedl syndrome (BBS) [17,18]. The *tubby* mutation is a G-to-T transversion that abolishes the donor splice site in the penultimate exon (exon 11), resulting in an aberrant transcript [19]. This leads to the substitution of the tubby C-terminal 44 amino acids with 24 different residues encoded by the intron. The spontaneous mutation in the *tubby* mouse (*Tub*^*tub/tub*^) appears to cause a loss of function, as targeted disruption of the *tub* gene gives a similar phenotype [20]. The original study on *tubby* mice found moderate and progressive hearing loss, and a moderate retinal degeneration [16]. A modifier gene *Mtap1a* modulates the severity of hearing loss and retinal degeneration in the *tubby* mutant mice [21,22]. One interesting feature of the *tubby* mutant that is shared with the *Tulp1* knockout mouse is the extracellular accumulation of rhodopsin-laden vesicles in the interphotoreceptor space surrounding the photoreceptor inner segments, which peaks at around 17 to 21 days of age when rhodopsin is rapidly synthesized to build up the outer segments [16]. The vesicles are relatively uniform in size averaging 0.1 to 0.2 μm in diameter and bounded by a single membrane. This distinct phenotype is also seen in transgenic mice carrying a C-terminal rhodopsin mutation known to affect specifically the trafficking of rhodopsin to the outer segments [23]. It was, therefore, hypothesized that the extracellular vesicle accumulation might be a hallmark of defect in the directional transport of nascent rhodopsin to the outer segments, thus implying a role for tubby and TULP1 in rhodopsin trafficking in photoreceptors [9]. In further support of this hypothesis, mice doubly mutant for *tubby* and *Tulp1* have a much more severe retinal phenotype than either mutant alone, manifesting a complete failure of rhodopsin trafficking and outer segment formation, and rapid cell death. These data would appear to suggest that tubby may function synergistically with TULP1 in a pathway that facilitates rhodopsin trafficking to the outer segments [9]. Differing from TULP1*,* which is photoreceptor-specific, tubby has a wider range of expression but appears enriched in neuronal tissues.

Based on a structure-directed approach, it has been proposed that tubby-like proteins are a unique family of bipartite transcription factors [14,24]. The molecular architecture of tubby-like proteins is seen as well suited for a function in transcriptional modulation. There is the nuclear localization signal at the N terminus of tubby, an ability of the N-terminal domain to activate transcription when fused to a DNA binding motif and the ability of the conserved C-terminal tubby domain to bind DNA and phosphatidylinositol 4, 5-bisphosphate (PIP_2_). That the tubby domain binds specifically to PIP_2_ has been well established [25] but the transcriptional target genes of tubby have remained unknown. In another series of studies, tubby was proposed to be a MerTK ligand that mediates phagocytosis of the photoreceptor outer segments by retinal pigment epithelia [26]. These findings represented advances in the molecular dissection of tubby function, but how they relate to the *in vivo* role of tubby and the *tubby* mutant phenotype has been less clear. In this study, we examined the subcellular distribution of a number GPCRs and show that tubby is essential for GPCR trafficking in the neuronal and sensory cilia.

## Methods

### Animals

All animal care and procedures were approved by Animal Care and Use Committees at the Dean A. McGee Eye Institute and the National Eye Institute. Mice were maintained in an animal facility under a 12-h light/12-h dark lighting cycle. Genotyping for *tubby* mutation was based on a published protocol [27]. WT and *tubby* mutant mice at 1 month of age were used for analysis of the brain tissue, and mice at 1 month and at 12 days were used to analyze the retinal tissues.

### Generation of tubby antibody

A His-tagged fusion protein encompassing the N-terminal 200 amino acid residues of mouse tubby protein was expressed in *E. coli*, purified and used to generate a polyclonal antibody in rabbit. The antibody was affinity-purified.

### Immunoblotting analysis

Mice were euthanized and their retinas and brain were dissected out. Tissues were homogenized in RIPA buffer, boiled in Laemmli buffer and separated on 10% SDS-PAGE gels. Proteins were blotted to polyvinylidene difluoride (PVDF) membrane by electrotransfer. After blocking with 5% non-fat milk, the membranes were incubated with primary antibodies overnight at room temperature. After washing, membranes were incubated with peroxidase-conjugated secondary antibodies. SuperSignal® West Pico Chemiluminescent Substrate (Thermo Fisher Scientific, Rockford, IL, USA) was used for detection. For normalization, protein samples were separated on standard SDS-PAGE and probed with an anti-actin antibody.

### Immunofluorescence

For immunofluorescence, eyes were enucleated, placed in fixative and their anterior segments and lens were removed. Brains were placed directly in a fixative containing 2% paraformaldehyde in phosphate buffered saline (PBS) for a total duration of 1.5 to 2 hours. Tissues were embedded in 3% agarose and sectioned at 75-μm thickness using a vibratome. Sections were collected into PBS buffer and remained free floating for the duration of the immunostaining process. For staining neuronal primary cilia in the brain, tissue sections were subjected to heat antigen retrieval at 60°C in PBS overnight. Prior to incubating with primary antibodies, sections were exposed to 50 mM NaCNBH_3_ to quench background fluorescence and blocked in 5% goat serum/PBS. All antibody incubations were carried out for 16 to 24 hours at ambient temperature. Cell nuclei were counter stained blue by 4′,6-diamidino-2-phenylindole (DAPI). The sections were viewed and photographed on a laser scanning confocal microscope (model TCS SP2; Leica Microsystems, Wetzlar, Germany). Multiple consecutive focal planes (Z-stack), spaced at 0.5-μm intervals, were captured.

Primary antibodies used were anti-GRK1 (MA1-720, ABR), anti-RP1 (a gift of Dr. Eric Pierce), anti-ACIII (sc-588; Santa Cruz Biotechnology, Santa Cruz, CA, USA), anti-SSTR3 (ss-830, Biotrend Chemicals, Destin, FL, USA), anti-MCHR1 (sc- 5534; Santa Cruz Biotechnology), anti-Htr6 (NBP1-46557, Novus Biologicals, Littleton, CO, USA), anti-mOR28 (NB110-75089, Novus Biologicals) and anti-rootletin [28]. Secondary antibodies included Alexa Fluor 488-, 546- and 647- conjugated antibodies (Invitrogen, Grand Island, NY, USA), and Cy3-conjugated donkey anti-goat IgG and Cy3-conjugated donkey anti-rabbit IgG (Jackson ImmunoResearch Laboratories, Inc, West Grove, PA, USA).

### RNA isolation, cDNA synthesis and real-time quantitative PCR (qPCR)

Total RNA was isolated from age and genetic background-matched WT and *tubby* mouse brains (at one month of age) using the TRIZOL reagents (Life Technologies, Carlsbad, CA, USA). RNA concentration was measured with NanoDrop spectrophotometer (Thermo Scientific, Wilmington, DE, USA) at a wavelength of 260 nm. cDNA synthesis was primed with oligo (dT)_20_ using Invitrogen SuperScript First-Strand Synthesis System (Life Technologies, Carlsbad, CA, USA). qPCR was carried out on an ABI 7900HT system (Life Technologies). Predesigned TaqMan Gene Expression Assays were purchased from Life Technologies with the following assay IDs: *Sstr3*, Mm00436695_s1; *Mchr1*, Mm00653044_m1; Eif2s3y, Mm00468995_g1; *Hprt1*, Mm01318747_g1; Rps26, Mm02601831_g1; *Tuba1a*, Mm00846967_g1. PCR reactions were carried out in triplicate in two independent reactions for each gene assayed, and the mean value was used to calculate fold-change of *Sstr3* and *Mchr1* in *tubby* vs. WT tissues, after normalizing against the geometric mean of the four housekeeping genes used as internal controls (*Hprt1, Eif2s3y, Rsp26, Tuba1a*) [29]. The comparative C_T_ method (the 2_T_^-ΔΔC^ method) was used for analyzing qPCR data [30].

## Results

### Mislocalization of rhodopsin and cone opsin in the *tubby* mice

**Figure 1:**
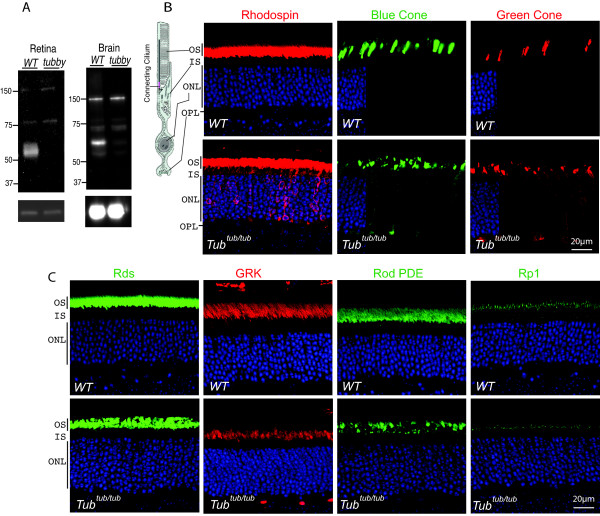
**Immunofluorescence analyses of the *tubby *mutant retina. (A)** The WT tubby protein migrates at approximately 60 kDa by immunoblotting, but tubby protein expression is absent in the mutant tissues indicating that the mutation is a functional *null* allele. The lower panel shows the actin immunoblotting results as a loading control. **(B)** Rhodopsin is localized primarily in photoreceptor outer segments in WT but is severely mislocalized to the cell body in the *tubby* mutant at one month of age. Similarly, blue and green cone opsins are normally localized to the cone outer segments in WT but are also mislocalized to the cell body and synaptic terminals in the *tubby* mutant retina. **(C)** In contrast to rhodopsin and cone opsins, peripherin/RDS and GRK, which are also integral membrane proteins, do not show mislocalization in the *tubby* mutant. Rod PDE and RP1 protein, which are membrane and cytoskeleton associated proteins, respectively, also manifest normal outer segment localization. IS, inner segment; ONL, outer (photoreceptor) nuclear layer; OPL, outer plexiform layer; OS, outer segment. Cell nuclei were counter stained blue by DAPI.

The aberrant *tubby* transcript is expressed at elevated levels in *tubby* mice [19]. To examine if any truncated forms of tubby protein may be expressed which may act dominantly and to compare the relative expression levels between different neural tissues, we generated a polyclonal antibody targeting the unique N-terminal domain of tubby and performed immunoblotting analysis of wild type (WT) and *tubby* mouse retina and brain extracts. As shown in Figure 1A, a prominent band at approximately 60 KDa was detectable in WT but not *tubby* mutant retina extracts. Similarly, a dominant band is detectable in the WT brain but absent in the mutant brain extract. No truncated form of tubby was found in the mutant tissue extracts. Since the tubby antibody targets the N-terminal domain and, therefore, would have been able to detect any truncated form of tubby, this observation indicates that the *tubby* mutation destabilizes the protein and behaves as a *null* allele. This finding largely replicates the result of a previous study [20]. Comparison with the actin loading control suggests that tubby is present in the retina at a much higher level than in the brain. Furthermore, tubby seems to be expressed as several variants as indicated by the broad banding pattern of the protein in the retina. The major brain tubby variant is of a larger size than that of the retina. The functional significance of this is currently unknown.

The photoreceptor outer segment is a modified cilium, where the visual pigment rhodopsin, a G protein-coupled receptor and other components of the phototransduction cascade are concentrated. The proximal end of the outer segment is linked to the cell body (inner segment) via a connecting cilium which is structurally homologous to the transition zone of motile or primary cilia [31] (Figure 1B). We assessed outer segment protein trafficking in the *tubby* mutant retina. Previously, we have suggested that tubby may function similarly to TULP1 in facilitating rod and cone opsin trafficking [7,9], based on the phenotype of the double *tubby/Tulp1* mutant mice. In the present study, we assessed the rod and cone visual pigments localization in photoreceptors. As shown in Figure 1B, rhodopsin and cone opsins, the visual pigments in cone photoreceptors, in WT retina are normally localized exclusively in the outer segments. In the mutant, however, a substantial fraction of rhodopsin is mislocalized in the inner segments and cell bodies. Similarly, the cone opsins are also partially distributed in the inner segments, the perinuclear region and the synaptic terminals. Because of its abundance in the outer segments, the visual pigment is also a structural constituent of the outer segments. Decreased transport of the visual pigment likely explains the outer segments being shorter in the mutant. Retention of rhodopsin and most outer segment proteins in the cell body could be a secondary phenomenon to the advanced stage of photoreceptor degeneration. To gain further evidence that visual pigment mislocalization was a primary defect, we expanded our studies to include mutant retinas at postnatal Day 12 and found that opsin is similarly mislocalized (data not shown). In contrast to the mislocalization of the rhodopsin and cone opsins, which are 7-pass transmembrane GPCRs, the localization of non-GPCR membrane proteins, such as peripherin/RDS and rhodopsin kinase (GRK), appear unaffected (Figure 1C). Other membrane associated proteins, such as PDE, and cytoskeleton associated protein, such as RP1, are also unaffected (Figure 1C). These data suggest that transciliary protein trafficking is not generally defective in the *tubby* mutant. Thus the *tubby* mutation appears to retard rhodopsin traffic through the connecting cilium. The retinal phenotype of *tubby* mutant is similar to that of the *Tulp1* mutant mice although somewhat milder.

### Lack of ciliary localization of neuronal GPCR in the brain of *tubby* mice

**Figure 2:**
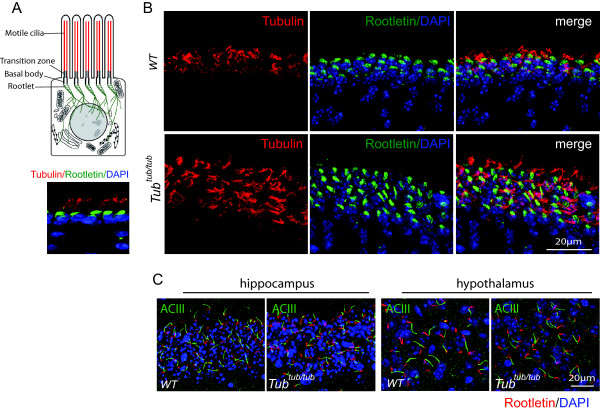
**Normal ciliogenesis and correct non-GPCR membrane protein trafficking in *tubby *mutant. (A)** The ependymal epithelia lining the lateral ventricles of WT mouse brain are multiciliated. These cilia are anchored by the ciliary rootlets. The ciliary axonemes are stained with an α-acetylated tubulin antibody (red) and the rootlets are highlighted by staining for rootletin (green). The upper image shows a schematic diagram and the lower image shows actual immunofluorescence. **(B)** In both WT and mutant tissues, ependymal epithelia appear to develop cilia of comparable length, abundance and organization. The ciliary rootlets (green) appear as a patch beneath the cilia as they are not resolved individually. **(C)** Neuronal primary cilia in the CA1 hippocampus and the paraventricular hypothalamus are visualized by staining for ACIII (green). Rootletin (red) serves as a marker for the ciliary base. In both regions, the appearance of ACIII staining pattern is similar between the WT and *tubby* mutant. Cell nuclei were counter stained by DAPI (blue).

Primary cilia present on the central nervous system (CNS) neurons function as signaling organelles. Signaling cascades are activated upon stimulation of G protein coupled receptors that activate adenylate cyclase type III (ACIII). The latter is also concentrated in the primary cilia. ACIII activation increases production of cAMP, which serves as a second messenger to mediate a diverse array of cellular processes. To our knowledge, at least three GPCRs have been shown to concentrate at the plasma membrane of the cilia in the brain, namely MCHR1, SSTR3 and serotonin receptor 6 (5HT6 receptor) [32-34]. We, therefore, set out to examine if cilia-directed trafficking of these receptors is compromised in the *tubby* mutant. In pilot studies, we were not able to label 5HT6 in neuronal cilia; hence, we focused our efforts on MCHR1 and SSTR3. We first assessed if lack of tubby caused a failure of ciliogenesis, that is, failure of the cilia to emerge as has been shown in a number of other mouse models of ciliopathy. The ependymal epithelia lining the brain ventricles are multi-ciliated. These motile cilia are anchored to ciliary rootlets, composed of rootletin, as in all types of cilia [28,35] (Figure 2A). By labeling with α-acetylated tubulin, we show comparable abundance and appearance of motile cilia (Figure 2B). We then used ACIII as a membrane protein marker for neuronal cilia [36]. As shown in Figure 2C, in two select regions of the mouse brain, the hippocampus and hypothalamus, ACIII clearly delineated the elongated primary cilia. Thus loss of tubby function does not appear to cause a generalized failure in ciliogenesis/maintenance, nor does it affect membrane protein trafficking to the cilia in general.

**Figure 3:**
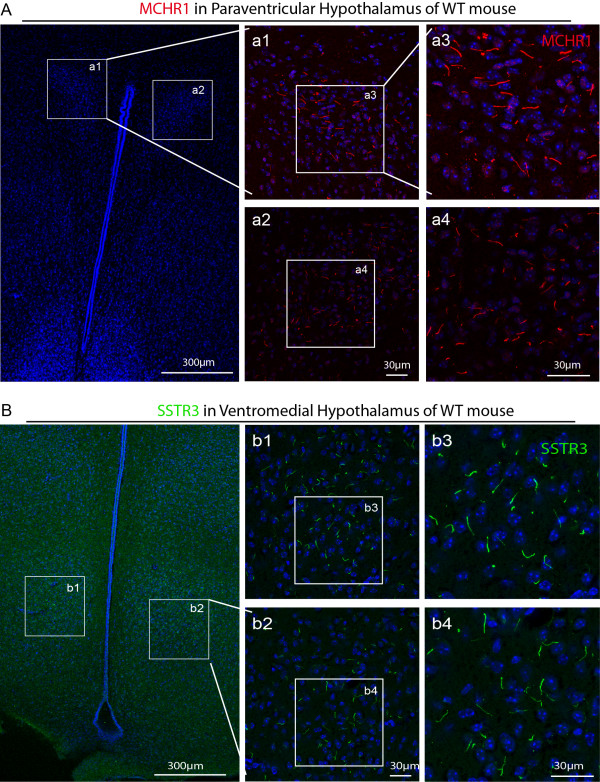
**SSTR3 and MCHR1 receptors in primary cilia of select brain regions. (A)** In the paraventricular hypothalamus region of the WT mouse brain, MCHR1 is seen concentrated in the neuronal cilia (red). **(B)** In the ventromedial hypothalamus region of the WT mouse brain, SSTR3 (green) is present in the neuronal primary cilia, thus validating the antibodies and the presence of these GPCRs in the cilia. Cell nuclei were counter stained blue by DAPI.

We next validated the ciliary localization of the GPCRs in the neuronal cilia that has been reported in the literature. As shown in Figure 3, MCHR1 and SSTR3 were both detected in the neuronal cilia in the hypothalamus region of the WT mice. Within the hypothalamus, SSTR3 was particularly prominent in the ventromedial and arcuate regions, while MCHR1 was primarily found in the paraventricular region, in agreement with the expression of *tubby* mRNA in hypothalamus [19]. Both receptors were also detected in many areas of the brain. In the hippocampus, MCHR1 and SSTR3 appeared in a complementary pattern with MCHR1, being particularly strong in CA1 but weak in CA3, and SSTR3 displaying the opposite pattern (data not shown). These data confirm the previously published finding that these GPCRs are concentrated in the neuronal primary cilia.

**Figure 4:**
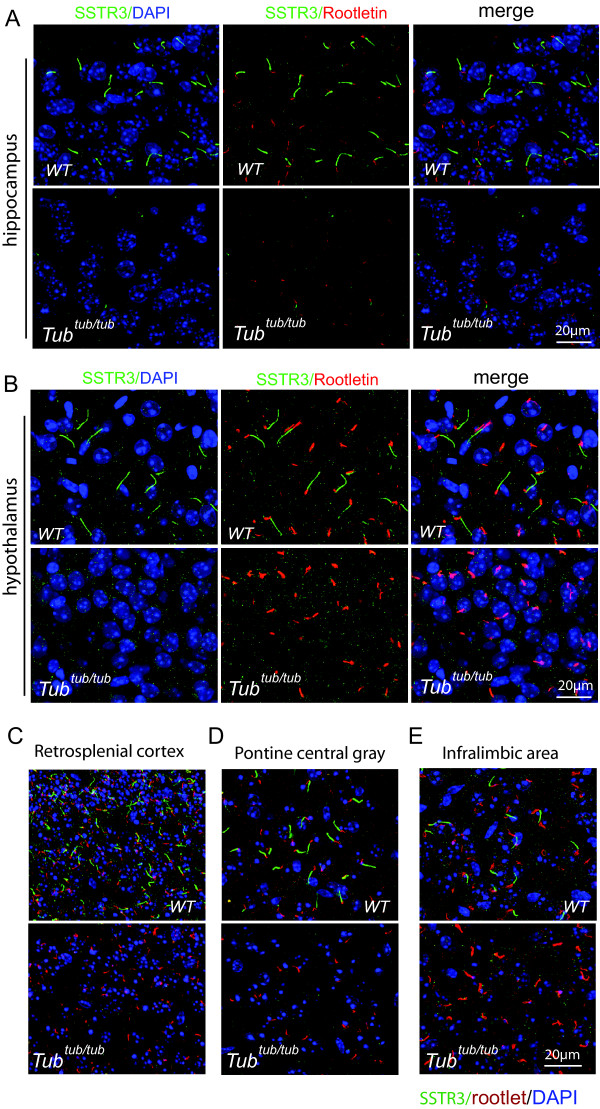
**Defective ciliary targeting of SSTR3 in the *tubby *mutant.** In both hippocampus **(A)** and in the hypothalamus **(B)** of the WT brain, SSTR3 (green) labels neuronal cilia strongly in the WT tissues (upper panels) but largely fail to label the cilia in the mutant (lower panels). The retrosplenial cortex **(C)**, pontine central gray **(D)** and infralimbic area **(E)** are three other brain regions where SSTR3 labeling of the neuronal cilia could be readily detected in the WT mouse. In all these regions, SSTR3 appear absent in the mutant. Rootletin staining (red) serves as a marker for the ciliary base. Cell nuclei were counter stained blue by DAPI.

We then compared the WT and the *tubby* mouse brains with respect to GPCR localization to the cilia. As shown in Figure 4A,B, in both the hippocampus and the hypothalamus regions of the *tubby* brain, SSTR3 is essentially undetectable or greatly diminished. The intensity of SSTR3 labeling is not uniform and shows considerable variability among different brain regions. Aside from the CA3 region of the hippocampus, additional regions of the mouse brain, such as the retrosplenial cortex, pontine central gray and infralimic area, also appeared to manifest a higher level of SSTR3 expression in the cilia. These regions, therefore, were carefully examined and compared between the WT and *tubby* mutant tissues. In all instances, we found the *tubby* mutant to have lost SSTR3 labeling of the primary cilia (Figure 4C). These data suggest that the SSTR3 receptor trafficking to neuronal primary cilia is generally disrupted in the *tubby* mutant.

**Figure 5:**
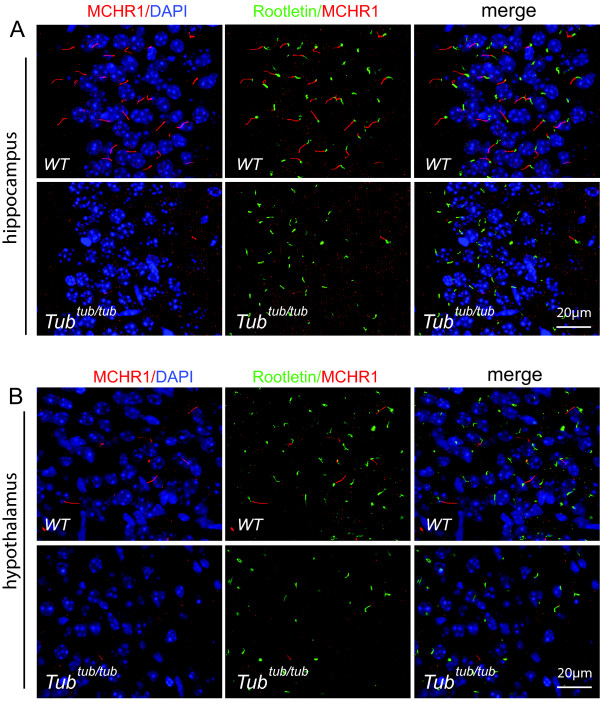
**Defective ciliary targeting of MCHR1 in the neuronal cilia of the *tubby *mutant.** MCHR1 (red) is found in neuronal cilia in WT (upper panels) but is lost from cilia in the mutant (lower panels) in both hippocampus **(A)** and in the hypothalamus **(B)**. Rootletin staining (green) serves as a marker for the ciliary base. Cell nuclei were stained (blue) with DAPI.

We obtained similar findings for the MCHR1 receptor (Figure 5). In both the hippocampus (Figure 5A) and hypothalamus (Figure 5B), MCHR1 is specifically localized in the neuronal cilia of the WT mouse brain but it is largely absent from the *tubby* mutant. As shown in Figure 5, the loss of neuronal cilia labeling for MCHR1 in the mutant is not accompanied by an increased ectopic labeling for the protein, suggesting either that the protein is destabilized or that the level of dispersed receptor protein is below the threshold of detection in our assay.

**Figure 6:**
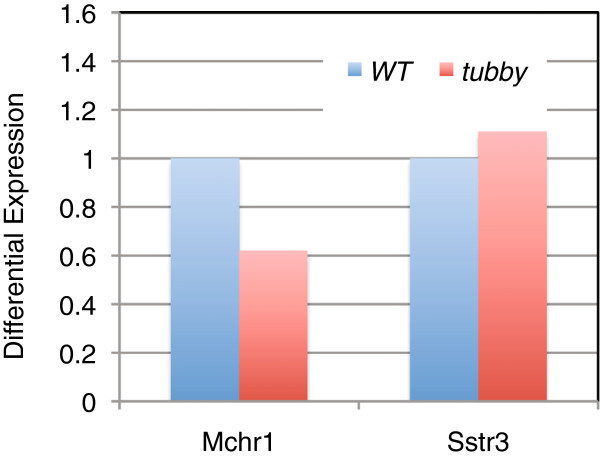
**Estimation of *Sstr3 *and *Mchr1 *transcript levels by qPCR.** Real time reverse transcription-quantitative PCR amplification of *Sstr3* and *Mchr1* mRNA from total brain RNA isolated from one pair of 20-day-old WT and *tubby* mutant mice using the TaqMan assay. Results shown are the means of triplicate assays from two independent RT-PCR experiments, normalized against the geometric mean of four genes that served as internal controls (*Hprt1, Eif2s3y, Rsp26, Tuba1a*). *Mchr1* and *Sstr3* transcripts are found at 62% and 115% of the WT levels, respectively.

As tubby could potentially serve as a transcriptional factor, an alternative explanation for the loss of receptors from the neuronal cilia could be a loss of transcription of those genes. We, therefore, investigated whether *Sstr3* and *Mchr1* mRNAs were absent or severely reduced in the *tubby* mouse brain. By reverse transcription and qPCR analysis, *Sstr3* mRNA in *tubby* appeared slightly higher than that of the WT, whereas *Mchr1* mRNA was found at approximately 60% of the WT levels (Figure 6). In neither case would loss of transcription appear to have taken place as a result of the *tubby* mutation, nor could that be an explanation for the disappearance of the receptors at the cilia.

### Role of tubby in the olfactory sensory epithelia

**Figure 7:**
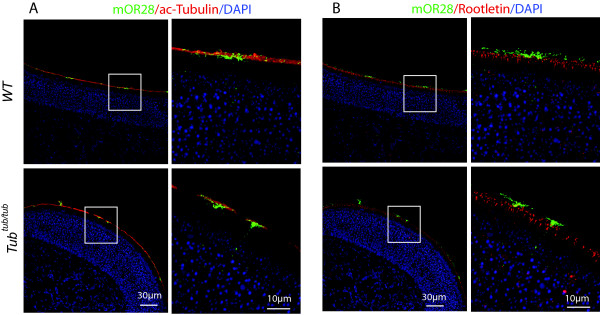
**Odorant receptors are trafficked normally to the cilia of olfactory sensory neurons in *tubby *mice.** Localization of the odorant receptor mOR28 in the olfactory sensory cilia appears indistinguishable between WT and *tubby* mutant. **(A)** mOR28 (green) double labeled with α-acetylated tubulin (red) which highlight the ciliary axoneme. In both WT and mutant tissues, mOR28 appear localized to the cilia layer with weaker signal appearing in the neuronal soma in both genotypes. **(B)** mOR28 (green) double labeled with rootletin (red). In both WT and mutant tissues, mOR28 is localized distal to rootletin, as would be expected for a ciliary localization.

The ciliated olfactory sensory neurons deploy a large repertoire of receptors, which are GPCRs, to their cilia for efficient sensing of environmental odorants. A previous study on *Bbs2* and *Bbs4* mutants has suggested that lack of these BBS proteins leads to a defect in the distribution odorant receptors in the olfactory cilia [37]. We examined if tubby might also be required for the ciliary targeting of odorant receptors. We isolated WT and *tubby* mutant olfactory epithelia and stained for mOR28, one of the odorant receptors, along with other ciliary markers. As shown in Figure 7, the development and maintenance of olfactory sensory cilia appear comparable between WT and the *tubby* mutant. In the mutant, the mOR28 receptor is distributed to the distal portion of the cilia in a manner that is indistinguishable from the WT, and there is no evidence that the receptor protein is ectopically retained within the cell body. These data suggest that tubby function appears dispensable for the correct trafficking of odorant receptors. Thus the repertoire of GPCRs that are critically dependent on BBS proteins for transciliary trafficking and those requiring tubby function appear to overlap but are not identical.

## Discussion

In this study we show that in the absence of tubby, a number of 7-pass transmembrane proteins that function as GPCRs are not properly transported to the primary or sensory cilia. In the brain, we have demonstrated that tubby is required for the transciliary trafficking of two GPCRs, MCHR1 and SSTR3. In multiple brain regions where these GPCRs are found to concentrate in the cilia in WT mice, we show that the same GPCRs are diminished or extinguished from the neuronal primary cilia of *tubby* mice. By co-labeling with α-acetylated tubulin for ciliary axoneme and with the ciliary membrane protein ACIII, we show that ciliogenesis or maintenance appear unaffected in the *tubby* mutant. Therefore, the primary defect in the tubby *mutant* is a block in transport of select GPCRs to the neuronal primary cilia. Tubby is not essential for all GPCR trafficking in neuronal cilia, however. In our study, we found that the odorant receptor mOR28, which is a GPCR that interacts with odorant molecules, remains correctly localized to the distal cilia of olfactory epithelial cells. The entire repertoire of GPCRs that requires tubby protein for trafficking is unlikely to be limited to just MCHR1 and SSTR3. As more cilia restricted GPCRs in the brain are being discovered and tested in the *tubby* mutant, a more complete list of tubby-dependent GPCR will emerge, which in turn, may help define precisely how tubby functions in a ciliary trafficking pathway.

In the *tubby* mutant retina, photoreceptor cells accumulate rhodopsin and cone opsins ectopically in the cell body, accompanied by shortened outer segments. Mislocalization of rhodopsin can occur as a secondary phenomenon to retinal degeneration as discussed previously [9]. On the other hand, most retinal degeneration mouse models, in which the affected gene does not function in rhodopsin or ciliary trafficking, do not show overt opsin mislocalization when the photoreceptor layers are largely still intact. For example, in the rhodopsin T17M transgenic mice [9] and in the P23H transgenic mice and rats [38-40], opsin correctly localize to the outer segments. In contrast, in the rhodopsin P347S mutant, which affects a known C-terminal trafficking signal [41,42], opsin is prominently mislocalized early on [23]. In the *tubby* mutant, opsin mislocalization is likely to be a primary defect since we observed rhodopsin mislocalization at an early time point (postnatal Days 12 and 30) while the photoreceptors are still relatively intact and when trafficking of most other membrane proteins remains normal. Furthermore, *tubby* mice accumulate extracellular vesicles at early postnatal ages which had been shown to be evidence of aberrant rhodopsin trafficking [9]. We believe, therefore, that rhodopsin mislocalization is a primary defect in the *tubby* mutant photoreceptor, much like that observed in the related *Tulp1* mutant mouse in previous studies [9]. The defect in the photoreceptors is limited to GPCRs, rather than a generalized failure of targeting proteins to the outer segments.

In the sensory hair cells of the cochleas, loss of tubby function apparently has a negative impact as well as indicated by the hearing loss in the *tubby* mice. We hypothesize that the primary defect in the cochlea hair cells might also originate from a defective protein trafficking along the kinocilia. It remains unclear, however, what GPCRs might be candidates for tubby-dependent transport machinery in cochlear hair cells.

Both the *tubby* mouse phenotype and the cellular defect described in this study appear remarkably similar to those found in the mouse models of BBS. Both models develop obesity, retinitis pigmentosa and hearing loss. Although human BBS patients often show polydactyly, neither mouse models do so, reflecting again the similarity of the two disease models. Human BBS also manifests mental retardation. In BBS2 and BBS4 mouse mutants, both SSTR3 and MCHR1 receptors were found to be absent from neuronal primary cilia [37]. MCHR1 mediated signaling in the hypothalamus regulates food intake and energy homeostasis [43], and the disruption of its normal localization in the cilia could underlie at least in part the obesity phenotype of the *tubby* mice. SSTR3 signaling in the cilia of hippocampal neurons appears to couple to ACIII. Disruption of somatostatin signaling in the hippocampus, as revealed in the study of *Sstr3* knockout mice, leads to lower cAMP levels in the hippocampus and a defect in novel object learning [44]. This phenotype is similar to that of ACIII knockout mice [45]. Many BBS proteins have been found to localize to the base of cilia. In the case of tubby like proteins, we have previously shown that TULP1 is diffusely distributed throughout the photoreceptor cell body [9]. We were unable to pinpoint tubby protein in the proximity of cilia either in photoreceptors or in CNS neurons (data not shown). A previous work by Ikeda *et al.* also found a diffuse pattern of tubby staining in the retina. Thus tubby and TULP1 seem to differ from TULP3, which localizes to the base or ciliary shaft in cultured cells [11,15]. It is possible that tubby will behave differently in cultured neurons vs. *in vivo* tissues, or that tubby localizes to the cilia under specific conditions. Further experiments will be needed to clarify these points. A human disease attributable to a *tubby* mutation has not been identified, but BBS is a plausible candidate.

How tubby-like proteins perform their functions remain incompletely understood. Available data together indicate a role for tubby proteins at the cilia. In the case of tubby, TULP1 and TULP3, ciliary trafficking of GPCRs is likely to be a major aspect of their ciliary functions, whereas TULP2 has been implicated in ciliogenesis [12]. A recent study provides important insights into how tubby proteins may execute this process [1,15]. In that study, it was found that TULP3 interacts with IFT-A particles to traffic membrane receptors to cilia, and modulation by phosphoinositide binding is also required for this process. Furthermore, the IFT-binding sequence in the divergent N-terminal domain is also present in tubby and TULP2, suggesting that other *tubby* family members may also interact with IFT-A. Thus, a functional interaction with IFT particles during ciliary trafficking may be generally applicable to mechanisms of action by tubby-like proteins. It remains to be determined how the ciliary trafficking role of tubby-like proteins might reconcile with the proposed transcription modulator model [14]. In that model, tubby associates with plasma membranes where PIP_2_ contents are high. Upon G protein signaling and hydrolysis of PIP_2_ by phospholipase C, tubby dissociates from plasma membrane and translocates to the nucleus where it binds DNA and modulates gene transcription. It is possible that tubby engages in a similar process, getting on and off plasma membranes depending on the phosphoinositide content along the route of ciliary trafficking. In this regard, it is interesting to note that PIP_2_ phosphatases, INPP5E and INPP5F, are localized in the cilia and their functional deficits are associated with ciliopathy [46-49], thus creating a potential gradient of PIP_2_ physiologically at the cilia-plasma membrane junction. As to whether and how tubby-like proteins modulate gene transcription or serve as a ligand for phagocytosis will await further studies.

## Conclusions

Tubby protein, and the tubby-like family of proteins in general, are involved in transciliary trafficking of select GPCRs. The GPCR trafficking defects we identified could largely explain the phenotype of the *tubby* mutant mice.

## Abbreviations

BBS: Bardet-Biedl syndrome; DAPI: 4^′^,6-diamidino-2-phenylindole; GPCR: G protein-coupled receptors; MCHR1: Melanin concentrating hormone receptor 1; PBS: Phosphate-buffered saline; PIP_2_: Phosphatidylinositol 4, 5-bisphosphate; PVDF: Polyvinylidene difluoride; SSTR3: Somatostatin receptor 3; WT: Wild type.

## Competing interests

The authors declare that they have no competing interests.

## Authors’ contributions

XS participated in the study design, carried out the full series of the experiments and helped to draft the manuscript. JH carried out the initial immunolabeling experiments. OB performed immunoblotting and RNA extraction. XC maintained the *tubby* mutant line, performed genotyping, procured and supplied tissues for experiments, and helped to revise the manuscript. JM provided the *tubby* mouse line and helped to revise the manuscript. TL conceived of the study, and participated in its design and coordination, and wrote the manuscript. All authors read and approved the final manuscript.

## References

[B1] Mukhopadhyay S Jackson PK The tubby family proteins Genome Biol 2011 12 225 10.1186/gb-2011-12-6-225 21722349 PMC3218838

[B2] Carroll K Gomez C Shapiro L Tubby proteins: the plot thickens Nat Rev Mol Cell Biol 5 55 63 14708010 10.1038/nrm1278

[B3] North MA Naggert JK Yan Y Noben-Trauth K Nishina PM Molecular characterization of TUB, TULP1, and TULP2, members of the novel tubby gene family and their possible relation to ocular diseases Proc Natl Acad Sci U S A 1997 94 3128 3133 10.1073/pnas.94.7.3128 9096357 PMC20333

[B4] Noben-Trauth K Naggert JK North MA Nishina PM A candidate gene for the mouse mutation tubby Nature 1996 380 534 538 10.1038/380534a0 8606774

[B5] Ikeda S He W Ikeda A Naggert JK North MA Nishina PM Cell-specific expression of tubby gene family members (tub, Tulp1,2, and 3) in the retina Invest Ophthalmol Vis Sci 1999 40 2706 2712 10509669

[B6] Hagstrom SA North MA Nishina PL Berson EL Dryja TP Recessive mutations in the gene encoding the tubby-like protein TULP1 in patients with retinitis pigmentosa Nat Genet 1998 18 174 176 10.1038/ng0298-174 9462750

[B7] Hagstrom SA Duyao M North MA Li T Retinal degeneration in tulp1−/− mice: vesiclular accumulation in the interphotoreceptor matrix Invest Ophthalmol Vis Sci 1999 40 2795 2802 10549638

[B8] Ikeda S Shiva N Ikeda A Smith RS Nusinowitz S Yan G Lin TR Chu S Heckenlively JR North MA Naggert JK Nishina PM Duyao MP Retinal degeneration but not obesity is observed in null mutants of the tubby-like protein 1 gene Hum Mol Genet 2000 9 155 163 10.1093/hmg/9.2.155 10607826

[B9] Hagstrom SA Adamian M Scimeca M Pawlyk BS Yue G Li T A role for the Tubby-like protein 1 in rhodopsin transport Invest Ophthalmol Vis Sci 2001 42 1955 1962 11481257

[B10] Nishina PM North MA Ikeda A Yan Y Naggert JK Molecular characterization of a novel tubby gene family member, TULP3, in mouse and humans Genomics 1998 54 215 220 10.1006/geno.1998.5567 9828123

[B11] Norman RX Ko HW Huang V Eun CM Abler LL Zhang Z Sun X Eggenschwiler JT Tubby-like protein 3 (TULP3) regulates patterning in the mouse embryo through inhibition of Hedgehog signaling Hum Mol Genet 2009 18 1740 1754 10.1093/hmg/ddp113 19286674 PMC2671991

[B12] Stolc V Samanta MP Tongprasit W Marshall WF Genome-wide transcriptional analysis of flagellar regeneration in Chlamydomonas reinhardtii identifies orthologs of ciliary disease genes Proc Natl Acad Sci U S A 2005 102 3703 3707 10.1073/pnas.0408358102 15738400 PMC553310

[B13] Bell CG Benzinou M Siddiq A Lecoeur C Dina C Lemainque A Clement K Basdevant A Guy-Grand B Mein CA Meyre D Froguel P Genome-wide linkage analysis for severe obesity in French Caucasians finds significant susceptibility locus on chromosome 19q Diabetes 2004 53 1857 1865 10.2337/diabetes.53.7.1857 15220211

[B14] Santagata S Boggon TJ Baird CL Gomez CA Zhao J Shan WS Myszka DG Shapiro L G-protein signaling through tubby proteins Science 2001 292 2041 2050 10.1126/science.1061233 11375483

[B15] Mukhopadhyay S Wen X Chih B Nelson CD Lane WS Scales SJ Jackson PK TULP3 bridges the IFT-A complex and membrane phosphoinositides to promote trafficking of G protein-coupled receptors into primary cilia Genes Dev 2010 24 2180 2193 10.1101/gad.1966210 20889716 PMC2947770

[B16] Heckenlively JR Chang B Erway LC Peng C Hawes NL Hageman GS Roderick TH Mouse model for Usher syndrome: Linkage mapping suggests homology to Usher type I reported at human chromosome 11p15 Proc Natl Acad Sci U S A 1995 92 11100 11104 10.1073/pnas.92.24.11100 7479945 PMC40579

[B17] Zhang Q Nishimura D Seo S Vogel T Morgan DA Searby C Bugge K Stone EM Rahmouni K Sheffield VC Bardet-Biedl syndrome 3 (BBs3) knockout mouse model reveals common BBS-associated phenotypes and BBs3 unique phenotypes Proc Natl Acad Sci U S A 2011 108 20678 20683 10.1073/pnas.1113220108 22139371 PMC3251145

[B18] Sheffield VC The blind leading the obese: the molecular pathophysiology of a human obesity syndrome Trans Am Clin Climatol Assoc 2010 121 172 181 discussion 181–182 20697559 PMC2917141

[B19] Kleyn PW Fan W Kovats SG Lee JJ Pulido JC Wu Y Berkemeier LR Misumi DJ Holmgren L Charlat O Woolf EA Tayber O Brody T Shu P Hawkins F Kennedy B Baldini L Ebeling C Alperin GD Deeds J Lakey ND Culpepper J Chen H Glücksmann-Kuis MA Carlson GA Duyk GM Moore KJ Identification and characterization of the mouse obesity gene tubby: a member of a novel gene family Cell 1996 85 281 290 10.1016/S0092-8674(00)81104-6 8612280

[B20] Stubdal H Lynch CA Moriarty A Fang Q Chickering T Deeds JD Fairchild-Huntress V Charlat O Dunmore JH Kleyn P Huszar D Kapeller R Targeted deletion of the tub mouse obesity gene reveals that tubby is a loss-of-function mutation Mol Cell Biol 2000 20 878 882 10.1128/MCB.20.3.878-882.2000 10629044 PMC85204

[B21] Ikeda A Zheng QY Zuberi AR Johnson KR Naggert JK Nishina PM Microtubule-associated protein 1A is a modifier of tubby hearing (moth1) Nat Genet 2002 30 401 405 10.1038/ng838 11925566 PMC2862212

[B22] Maddox DM Ikeda S Ikeda A Zhang W Krebs MP Nishina PM Naggert JK An allele of microtubule-associated protein 1A (Mtap1a) reduces photoreceptor degeneration in Tulp1 and Tub mutant mice Invest Ophthalmol Vis Sci 2012 53 1663 1669 10.1167/iovs.11-8871 22323461 PMC3339923

[B23] Li T Snyder WK Olsson JE Dryja TP Transgenic mice carrying the dominant rhodopsin mutation P347S: evidence for defective vectorial transport of rhodopsin to the outer segments Proc Natl Acad Sci U S A 1996 93 14176 14181 10.1073/pnas.93.24.14176 8943080 PMC19513

[B24] Boggon TJ Shan WS Santagata S Myers SC Shapiro L Implication of tubby proteins as transcription factors by structure-based functional analysis Science 286 2119 2125 10591637 10.1126/science.286.5447.2119

[B25] Quinn KV Behe P Tinker A Monitoring changes in membrane phosphatidylinositol 4,5-bisphosphate in living cells using a domain from the transcription factor tubby J Physiol 2008 586 2855 2871 10.1113/jphysiol.2008.153791 18420701 PMC2517197

[B26] Caberoy NB Zhou Y Li W Tubby and tubby-like protein 1 are new MerTK ligands for phagocytosis EMBO J 2010 29 3898 3910 10.1038/emboj.2010.265 20978472 PMC3020645

[B27] Maddatu T Naggert JK Allele-specific PCR assays for the tub and cpefat mutations Mamm Genome 1997 8 857 858 10.1007/s003359900594 9337402

[B28] Yang J Liu X Yue G Adamian M Bulgakov O Li T Rootletin, a novel coiled-coil protein, is a structural component of the ciliary rootlet J Cell Biol 2002 159 431 440 10.1083/jcb.200207153 12427867 PMC2173070

[B29] Vandesompele J De Preter K Pattyn F Poppe B Van Roy N De Paepe A Speleman F Accurate normalization of real-time quantitative RT-PCR data by geometric averaging of multiple internal control genes Genome Biol 2002 3 RESEARCH0034 12184808 10.1186/gb-2002-3-7-research0034 PMC126239

[B30] Schmittgen TD Livak KJ Analyzing real-time PCR data by the comparative C(T) method Nat Protoc 2008 3 1101 1108 10.1038/nprot.2008.73 18546601

[B31] Besharse JC Horst CJ Ciliary and Flagellar Membranes The photoreceptor connecting cilium: a model for the transition zone 1990 Plenum, New York 389 417

[B32] Berbari NF Johnson AD Lewis JS Askwith CC Mykytyn K Identification of ciliary localization sequences within the third intracellular loop of G protein-coupled receptors Mol Biol Cell 2008 19 1540 1547 10.1091/mbc.E07-09-0942 18256283 PMC2291422

[B33] Brailov I Bancila M Brisorgueil MJ Miquel MC Hamon M Verge D Localization of 5-HT(6) receptors at the plasma membrane of neuronal cilia in the rat brain Brain Res 2000 872 271 275 10.1016/S0006-8993(00)02519-1 10924708

[B34] Handel M Schulz S Stanarius A Schreff M Erdtmann-Vourliotis M Schmidt H Wolf G Hollt V Selective targeting of somatostatin receptor 3 to neuronal cilia Neuroscience 1999 89 909 926 10.1016/S0306-4522(98)00354-6 10199624

[B35] Yang J Li T Focus on molecules: rootletin Exp Eye Res 2006 83 1 2 10.1016/j.exer.2005.10.013 16318850

[B36] Bishop GA Berbari NF Lewis J Mykytyn K Type III adenylyl cyclase localizes to primary cilia throughout the adult mouse brain J Comp Neurol 2007 505 562 571 10.1002/cne.21510 17924533

[B37] Berbari NF Lewis JS Bishop GA Askwith CC Mykytyn K Bardet-Biedl syndrome proteins are required for the localization of G protein-coupled receptors to primary cilia Proc Natl Acad Sci U S A 2008 105 4242 4246 10.1073/pnas.0711027105 18334641 PMC2393805

[B38] Olsson JE Gordon JW Pawlyk BS Roof D Hayes A Molday RS Mukai S Cowley GS Berson EL Dryja TP Transgenic mice with a rhodopsin mutation (Pro23His): a mouse model of autosomal dominant retinitis pigmentosa Neuron 1992 9 815 830 10.1016/0896-6273(92)90236-7 1418997

[B39] Liu X Wu T-H Stowe S Matsushita A Arikawa K Naash MI Williams DS Defective phototransductive disk membrane morphogenesis in transgenic mice expressing opsin with a mutated N-terminal domain J Cell Sci 1997 110 2589 2597 9372448 10.1242/jcs.110.20.2589

[B40] Green ES Menz MD LaVail MM Flannery JG Characterization of rhodopsin mis-sorting and constitutive activation in a transgenic rat model of retinitis pigmentosa Invest Ophthalmol Vis Sci 2000 41 1546 1553 10798675

[B41] Deretic D Puleo-Scheppke B Trippe C Cytoplasmic domain of rhodopsin is essential for post-Golgi vesicle formation in a retinal cell-free system J Biol Chem 1996 271 2279 2286 10.1074/jbc.271.4.2279 8567690

[B42] Sung CH Makino C Baylor D Nathans J A rhodopsin gene mutation responsible for autosomal dominant retinitis pigmentosa results in a protein that is defective in localization to the photoreceptor outer segment J Neurosci 1994 14 5818 5833 7523628 10.1523/JNEUROSCI.14-10-05818.1994 PMC6576989

[B43] Chen Y Hu C Hsu CK Zhang Q Bi C Asnicar M Hsiung HM Fox N Slieker LJ Yang DD Heiman ML Shi Y Targeted disruption of the melanin-concentrating hormone receptor-1 results in hyperphagia and resistance to diet-induced obesity Endocrinology 2002 143 2469 2477 10.1210/en.143.7.2469 12072376

[B44] Einstein EB Patterson CA Hon BJ Regan KA Reddi J Melnikoff DE Mateer MJ Schulz S Johnson BN Tallent MK Somatostatin signaling in neuronal cilia is critical for object recognition memory J Neurosci 2010 30 4306 4314 10.1523/JNEUROSCI.5295-09.2010 20335466 PMC3842454

[B45] Wang Z Phan T Storm DR The type 3 adenylyl cyclase is required for novel object learning and extinction of contextual memory: role of cAMP signaling in primary cilia J Neurosci 2011 31 5557 5561 10.1523/JNEUROSCI.6561-10.2011 21490195 PMC3091825

[B46] Bielas SL Silhavy JL Brancati F Kisseleva MV Al-Gazali L Sztriha L Bayoumi RA Zaki MS Abdel-Aleem A Rosti RO Kayserili H Swistun D Scott LC Bertini E Boltshauser E Fazzi E Travaglini L Field SJ Gayral S Jacoby M Schurmans S Dallapiccola B Majerus PW Valente EM Gleeson JG Mutations in INPP5E, encoding inositol polyphosphate-5-phosphatase E, link phosphatidyl inositol signaling to the ciliopathies Nat Genet 2009 41 1032 1036 10.1038/ng.423 19668216 PMC2746682

[B47] Jacoby M Cox JJ Gayral S Hampshire DJ Ayub M Blockmans M Pernot E Kisseleva MV Compere P Schiffmann SN Gergely F Riley JH Pérez-Morga D Woods CG Schurmans S INPP5E mutations cause primary cilium signaling defects, ciliary instability and ciliopathies in human and mouse Nat Genet 2009 41 1027 1031 10.1038/ng.427 19668215

[B48] Ishikawa H Thompson J Yates JR 3rd Marshall WF Proteomic analysis of mammalian primary cilia Curr Biol 2012 22 414 419 10.1016/j.cub.2012.01.031 22326026 PMC3298568

[B49] Luo N West C Murga-Zamalloa C Sun L Anderson RM Wells C Weinreb RN Travers JB Khanna H Sun Y OCRL localizes to the primary cilium: a new role for cilia in Lowe syndrome Hum Mol Genet 2012 21 3333 3344 10.1093/hmg/dds163 22543976 PMC3392109

